# Evaluation Method of Fatigue Life for Asphalt Pavement on the Steel Bridge Deck Based on the Inhomogeneous Poisson Stochastic Process

**DOI:** 10.3390/ma17040780

**Published:** 2024-02-06

**Authors:** Xunqian Xu, Guozhi Wan, Fengyi Kang, Shue Li, Wei Huang, Yu Li, Qi Li, Chen Lv

**Affiliations:** 1 School of Transportation and Civil Engineering, Nantong University, Nantong 226019, China; xunqian_xu@ntu.edu.cn (X.X.); gzwan_ntu@163.com (G.W.); seli_ntu@163.com (S.L.); yli_ntu@163.com (Y.L.); qli_ntu@163.com (Q.L.); clv_ntu@163.com (C.L.); 2 Nantong Highway Development Center, Nantong 226007, China; 3 Intelligent Transportation System Research Center, Southeast University, Nanjing 210096, China; seuwei_huang@163.com

**Keywords:** asphalt pavement, steel bridge deck, equivalent load spectrum, inhomogeneous Poisson stochastic process, damage estimation, fatigue life

## Abstract

The paving layer on the steel box girder bridge deck is widely used when constructing pavements for steel bridges. Owing to the orthotropic feature of steel decks, a transverse clapboard and rib can lead to a concentration of stress. Consequently, fatigue cracks are often identified in asphalt concrete pavement layers due to re-compaction caused by heavy vehicles. This study aims to derive an evaluation method of fatigue life for asphalt pavement based on the inhomogeneous Poisson stochastic process in view of the highly random and uncertain working conditions of layered composite structures. According to the inhomogeneous Poisson stochastic process, along with Miner’s fatigue damage accumulation theory and the linear elastic fracture mechanics theory, the fatigue life formula could be deduced. Meanwhile, fatigue experiments for asphalt concrete are designed to investigate the correlation between the theoretical formula and the actual fatigue damage life of the material. Compared with the test, the accuracy error is within 10%, which is better than other traditional methods. Therefore, the fatigue life prediction model could better reflect the loading order effect and the interaction between loads, providing a new path for the fatigue reliability design of steel bridge deck asphalt pavement.

## 1. Introduction

The large-span steel bridges, as the throat of the road network, are under continuous construction with the rapid development of the urban highway network. The paving layer on the steel box girder bridge deck has the main function of providing vehicles with a stable, smooth, and safe road surface. The surfacing layer needs to satisfy the requirements of high strength and durability, good abrasion and slip resistance, excellent high-temperature stability, low-temperature crack resistance, and waterproof ability. It also needs to have good deformation compatibility with the steel deck [1,2,3]. Orthotropic plate is not only used as the top flange of the steel box girder but also as the bottom plate of the asphalt paving layer. Because of the relatively small stiffness of the orthotropic plate structure, the paving layer is more complex than the asphalt mixture surface layer on an ordinary highway and is prone to being damaged by tensile force. Thus, the asphalt mixture pavement will crack due to re-compaction caused by the heavy vehicles.

In recent years, fatigue damage and cracking of asphalt mixtures have become major diseases affecting the use of bridges, especially large bridges, which are important hubs in the traffic network. Traffic parking maintenance will seriously affect traffic function and cause adverse social impacts [4,5,6]. Therefore, the research on the pavement performance of steel deck pavement systems, especially on the fatigue damage characteristics, has certain theoretical value and engineering significance [7,8,9]. Fatigue performance determines the service life length of steel-deck pavement structures under normal working conditions, and the accurate prediction of fatigue life is crucial to the structural strength design [10,11]. During the service period, the asphalt pavement of the steel bridge deck is often affected by the random vehicular load with variable amplitude. Tests showed that there were interactions between loads during the loading of variable-amplitude loads and large errors in the estimation of fatigue life if this effect was ignored [12,13]. The commonly used fatigue life estimation method adopted the linear fatigue damage theory, Miner’s theory, which has been widely applied in engineering practice because of its simplicity and practicability [14,15,16]. However, due to the significant simplification of the fatigue damage mechanism, the changes in internal structure, degree of damage, and damage accumulation and development processes caused by different loading processes cannot be considered. Accordingly, the calculation results would be presented with large errors [17,18]. In contrast, fuzzy fatigue damage theory takes the actual situation of the fatigue damage into account and considers that the load amplitude below the fatigue limit still contributes to the fatigue damage. Based on the results, the fuzzy region has been delineated, but the influence of load action sequence on the fatigue life estimation is still ignored, and the accuracy is thus not high [19,20]. By studying the stochastic process, the fatigue reliability of the distribution was found to agree with the Poisson stochastic process. The Poisson random distribution function has been introduced to describe the dynamic reliability of the parts and further proposes the probabilistic fatigue cumulative damage theory. However, the interaction effect between loads cannot be considered, so its estimation of structural fatigue reliability still has a bigger error [21].

Currently, most studies on the fatigue characteristics of the steel bridge deck asphalt pavement have ignored the loading sequence of cyclic loads and the influence between loads [22,23,24]. When a combination of the design method based on the s-n curve with fatigue cumulative damage theories has been adopted, it is necessary to more deeply study the important topic of fatigue cumulative damage law [25,26]. How to reasonably describe the load action sequence effect and the interaction between loads, establish a reasonable fatigue analysis model, and conduct more accurate analysis and judgment of the fatigue problems in the steel bridge deck asphalt pavement structure have become engineering problems that require to be addressed [27,28].

In this paper, a novel fatigue life estimation method was proposed, combining the Poisson stochastic process with associated theories and fully considering the interaction effect of loads on fatigue damage so as to bring it closer to the actual situation and improve the fatigue life estimation accuracy of the steel bridge deck asphalt pavement structure.

## 2. Poisson Stochastic Process Theory

Poisson stochastic process generally refers to the number of occurrence events over a certain interval of time $\left(t_{0},t\right)$, independent of the number of previous occurrences $t_{0}$, and the occurrence of events at each moment is random [29]. The mathematical model of load inhomogeneous Poisson strength function parameters was established by using the incomplete beta function, and thus the problem of path acquisition for the load micro-amplitude variation process has been addressed.

### 2.1. Inhomogeneous Poisson Stochastic Process Model

Based on the inhomogeneous Poisson stochastic process theory, the number of load occurrences over the period is assumed, and the following points are satisfied:

(1) $N\left(0\right)=0$, that is t=0, the occurrence times of load is 0.
(2) In any period $0<t_{1}<t_{2}<\cdots <t_{n}$, the occurrence of loads is independent of each other, and the occurrence times $N\left(t_{1}\right),N\left(t_{2}\right)-N\left(t_{1}\right),\cdots ,N\left(t_{n}\right)-N\left(t_{n-1}\right)$ of loads in this period are independent of each other.
(3) For any time t>0, and sufficiently small time interval Δt>0, there are

(1) $$P\left[N\left(t+\Delta t\right)-N\left(t\right)=1\right]=\lambda \left(t\right)\Delta t+ο\left(\Delta t\right)$$

(2) $$P\left[N\left(t+\Delta t\right)-N\left(t\right)\geq 2\right]=ο\left(\Delta t\right)$$

where, $P\left(\cdot \right)$ is the probability of occurrence.

(4) Based on the above conditions, within a small period Δt of time starting from time t=0, the probability of loads at all levels of the engineering structure is shown below.

(3) $$P\left[N_{i}\left(t+\Delta t\right)-N_{i}\left(t\right)=1\right]=\lambda \left(t\right)\Delta t+ο\left(\Delta t\right)$$

where $\lambda \left(t\right)$ is the inhomogeneous Poisson intensity function; $N_{i}$ is the cumulative frequency of i stage load; $ο\left(\Delta t\right)$ is a high-order infinitely small quantity of the occurrence probability of the load in a tiny time interval Δt.

The inhomogeneous Poisson strength coefficient can be determined by the linear model [30]

(4) $$\lambda \left(t\right)=\lambda \left(1+\alpha t\right)$$

where, α is the change rate parameter of the linear model, and λ is the corresponding homogeneous Poisson strength coefficient.

### 2.2. Parameter Derivation of the Poisson Stochastic Process

According to the theorem of large numbers, the homogeneous Poisson strength coefficient is defined as the ratio of the number of load cycles at all levels in the sample to the total number of load cycles in the sample, i.e.,

(5) $$\lambda_{i}=\frac{n_{i}}{N}$$

where, N is the total number of load cycles in the sample and $n_{i}$ is the number of load cycles at all levels in the sample.

**Figure 1:**
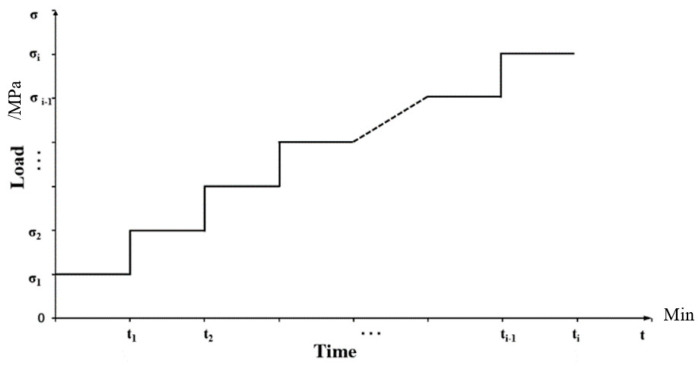
Loading process from low to high.

For the step spectrum, the step loading process is the easiest to implement and can be manually controlled. The hierarchical loading process can be regarded as a special random loading process with a fixed event rate. The continuous load spectrum can be fitted with a sufficiently dense step load spectrum. Additionally, the high-density step load spectrum can be used as the analysis object to determine the mathematical model of the coefficient of variation rates. Therefore, the inhomogeneous Poisson strength function of the graded load loading process can be analyzed and solved. The conditional, inhomogeneous Poisson strength function is obtained. The loading process, ranging from low to high, is shown in Figure 1. Similarly, the loading process goes from high to low.

At $0<t\leq t_{1}$, the occurrence probability of load $\sigma_{1}$ is always 1, and the occurrence probability of load $\sigma_{i\neq 1}$ is always 0; At $t_{1}<t\leq t_{2}$, the occurrence probability of load $\sigma_{2}$ is always 1, and the occurrence probability of load $\sigma_{i\neq 2}$ is always 0.

(6) $$\left\{\begin{array}{cc} \lambda_{i}\left(t\right)=\lambda_{i}\left(1+\alpha_{i}t\right)=1 & t_{i-1}<t\leq t_{i}\\ \lambda_{i}\left(t\right)=\lambda_{i}\left(1+\alpha_{i}t\right)=0 & t<t_{i-1}\;\mathrm{or}\;t>t_{i} \end{array}\right.$$

By deforming Equation (6) and introducing parameter β and step function $h\left(\beta \right)$, it can be deduced as

(7) $$\alpha_{i}=\frac{1}{t}{\left(\frac{N-n_{i}}{n_{i}}\right)}^{\beta_{i}}\cdot {\left(-1\right)}^{1+h\left(\beta_{i}\right)}$$

(8) $$h\left(\beta \right)=\left\{\begin{array}{cc} 1 & \beta \geq 0.5\\ 0 & \beta <0.5 \end{array}\right.$$

Then the inhomogeneous Poisson strength coefficient is

(9) $$\lambda_{i}\left(t\right)=\frac{n_{i}}{N}{\left(1+\frac{N-n_{i}}{n_{i}}\right)}^{\beta_{i}}\cdot {\left(-1\right)}^{1+h\left(\beta_{i}\right)}$$

LU et al. proposed a mathematical model to calculate the parameters in the load inhomogeneous Poisson strength function, as shown in Equation (10) [31].

(10) $$\beta \left(i\right)=\frac{\int_{0}^{x}x^{p_{j}-1}{\left(1-x\right)}^{q_{i}-1}dx}{B\left(p,q\right)}$$

where $\beta \left(i\right)$ is an incomplete beta function; $B\left(p,q\right)$ is the corresponding beta function, whose expression is $\int_{0}^{x}x^{p_{j}-1}{\left(1-x\right)}^{q_{i}-1}dx$. x is the ratio of the occurrence times of loads at all levels under the current number of cycles to the current number of cycles, i.e.,

(11) $$x=\frac{n_{i}\left(t\right)}{N\left(t\right)}$$

where, $N\left(t\right)$ is the current cycle time, and $n_{i}\left(t\right)$ is the occurrence times of loads at all levels under the current cycle times.

Parameters $p_{i},q_{i}$ reflect the distribution of beta function, and their value is related to the load distribution, which can be determined by Equation (12) [32].

(12) $$\left\{\begin{array}{c} p=\frac{{\left(\mu_{x}-u\right)}^{2}\left(\nu -\mu_{x}\right)-\sigma_{x}^{2}\left(\mu_{x}-u\right)}{\sigma_{x}^{2}\left(\nu -u\right)}\\ q=\frac{\left(\mu_{x}-u\right){\left(\nu -\mu_{x}\right)}^{2}-\sigma_{x}^{2}\left(\nu -\mu_{x}\right)}{\sigma_{x}^{2}\left(\nu -u\right)} \end{array}\right.$$

where $\mu_{x}$ is the mean of x, and $\sigma_{x}$ is the variance of x; when the upper and lower limits of beta distribution are u=0, ν=1 it becomes the standard beta distribution.

### 2.3. Calculation of Equivalent Load

All levels of loads have a certain probability of occurrence at all times t. Therefore, it is necessary to carry out equivalent processing of the time t load to solve the value of the time load, i.e.,

(13) $$\sigma_{e}\left(t\right)=\sum \sigma_{i}P_{i}\left(t\right)$$

where $P_{i}\left(t\right)$ is the probability of each level of load appearing at a time; $\sigma_{i}$ is the stress amplitude of each level of load; $\sigma_{e}$ is the equivalent load value at the time t.

## 3. Associated Damage Theory

There are potential neighborhood damage and associated damage between loads. Therefore, the amount of damage caused by the load cycle consists of two parts. The first is the apparent damage that does not affect the subsequent damage but reflects the level of the stress damage $D_{i}$. The second is the coupling damage, which affects the subsequent damage $D_{Ci}$. $D_{Ci}$ is the damage caused by the current stress in the spectrum load and affects the subsequent stress damage level. The value is related to the order of load action and the damage caused by the current stress. Therefore, the amount of damage from each load cycle can be expressed by Equation (14) as follows:

(14) $$D_{A}=D_{i}+D_{Ci}=\left\{\begin{array}{cc} D_{i} & \sigma <0.85\sigma_{r}\\ \left\{1+{\left(-1\right)}^{H\left(\sigma_{i+1}-\sigma_{i}\right)}\left[1-\exp\left(-{\left(\frac{\sigma_{i}-a}{b}\right)}^{2}\right)\right]\right\}D_{i} & \sigma \geq 0.85\sigma_{r} \end{array}\right.$$

(15) $$H\left(\sigma_{i+1}-\sigma_{i}\right)=\left\{\begin{array}{cc} 1 & \sigma_{i+1}-\sigma_{i}\geq 0\\ 0 & \sigma_{i+1}-\sigma_{i}<0 \end{array}\right.$$

where, $a=0.85\sigma_{r}$, $b=\left(0.05∼0.4\right)\sigma_{r}$, where $\sigma_{r}$ is the fatigue limit.

Apparent damage $D_{i}$ can be calculated by Equation (16) as follows:

(16) $$D_{i}=\frac{1}{N_{e}\left(t\right)}$$

where, $N_{e}\left(t\right)$ is the maximum number of cycles corresponding to the stress amplitude at the time t, which can be obtained through the S-N curve of the material [4].

## 4. Experiment

### 4.1. Raw Materials and Pavement Performances of Asphalt Concretes on Steel Bridge Decks

Epoxy Asphalt (EA) concrete is a common paving material used in long-span steel bridge decks. In this study, EA concrete was selected as the material for the pavement. The aggregate gradation and the optimum ratio of stone to oil for EA concrete are shown in Table 1.

The pavement performance, void fraction, dynamic stability, bending strength/strain, low temperature anti-crack property, rutting resistance, and water stability of asphalt concretes are examined according to the corresponding Chinese standard test methods of bitumen and bituminous mixtures for highway engineering (JTG E20-2011) [33]. Table 1 shows the measured void fraction, dynamic stability (60 °C and 70 °C), ultimate flexural strength/strain (−15 °C, 1 mm/min), indirect tensile strength (25 °C), indirect tensile strength after the freeze-thaw cycle (25 °C), linear contraction coefficient (15–−15 °C), and water stability. In addition, the technical standard requirements are provided in Table 2.

### 4.2. Fatigue Measurement of Asphalt Concrete Beam on Steel Plate

**Figure 2:**
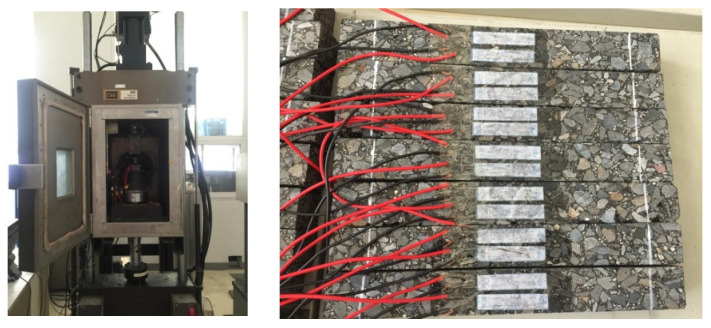
Test machine and beams.

The SHRP report provides a comprehensive evaluation and ranking of the degree of field simulations of different fatigue performance tests, the feasibility of each of the test methods, the feasibility of the test results, and correlations with the test results. It is considered that the cyclic bending test can represent the actual stress state of asphalt pavements adequately, and the results can be directly applied to engineering design. Therefore, this paper uses the bending fatigue test for beams to evaluate the fatigue damage performance of the asphalt pavement. For each asphalt mixture, board specimens of dimensions 380 mm × 100 mm × 50 mm were formed according to the optimal asphalt ratio, and the compactness of the rut board was controlled at 98% of the Marshall compactness. Three beams from each group are tested simultaneously, as shown in Figure 2.

**Figure 3:**
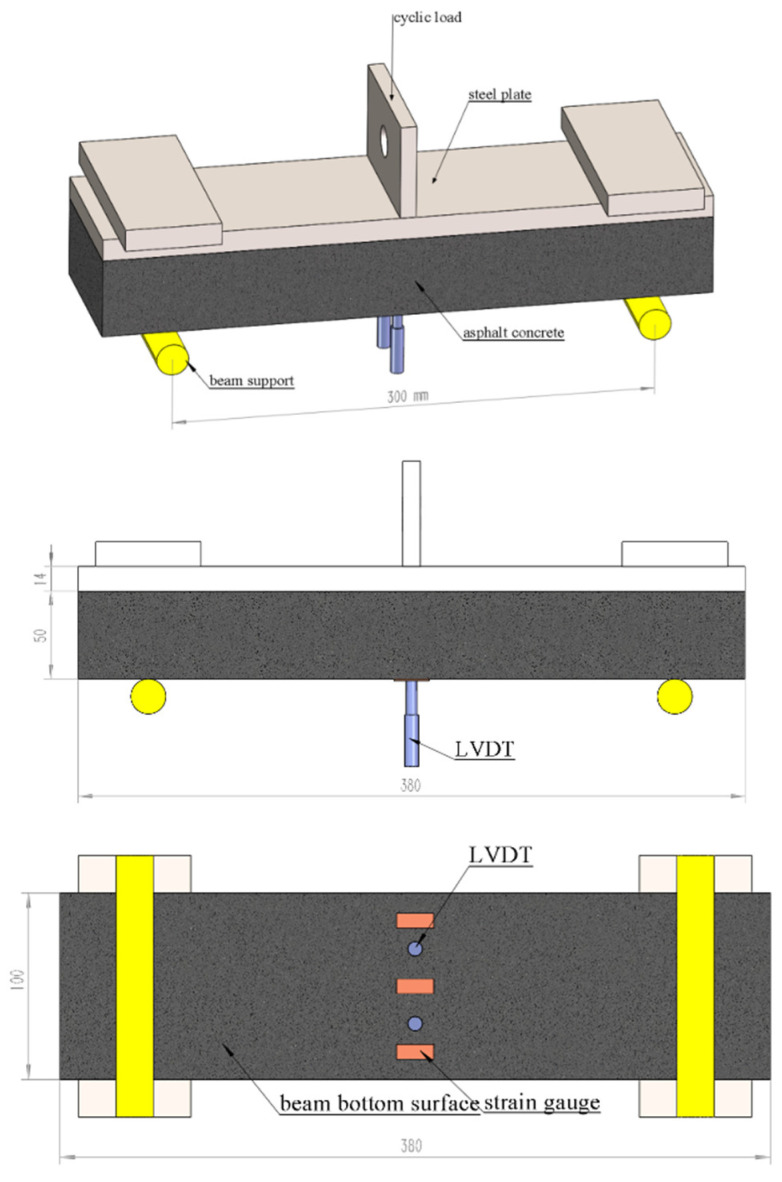
Schematic illustration of the fatigue test for asphalt concrete beam.

The trabecular specimen prepared with an epoxy asphalt mixture was used for a dynamic random loading fatigue test, as shown in Figure 3. The testing equipment (MTS Industrial Systems (China) Co., Ltd., Nanjing, China) consists of three parts: the test facility, the environment room, and the data control and acquisition system. The thickness of the steel bridge deck motherboard and asphalt pavement is t1 = 14 mm, t2 = 50 mm, and the width of the motherboard is W = 100 mm. The four stress amplitudes set in the test were S1 = 0.85 MPa, S2 = 0.75 MPa, S3 = 0.65 MPa, and S4 = 0.55 MPa. During the test, the load random loading program was set, and the dynamic cyclic loading was carried out continuously until the fatigue failure occurred. Thus, the test life of the specimen and the test cycle time of various stresses can be obtained, as shown in Table 3.

The test conditions for the temperature are 15 ± 0.5 °C, and the test specimens were retained for more than 4 h under the given temperature condition of ±0.5 °C. The fatigue load level can be deduced from the equivalent stress method, and it was calculated to be 12 kN and 16 kN by considering the action of standard axle loads of BZZ-100 kN and BJ-130 kN, respectively [3]. The continuous partial sine loading mode controlled by constant stress is adopted here, and the loading frequency is 10 ± 0.1 Hz. Under the target load level, when longitudinal cracks are formed on the surface of the specimen, it is considered that the specimen is damaged, and the loading test on the specimen is stopped.

For the fatigue tests, three strain gauges were attached at the bottom of the midspan of the composite beam to measure the strain, and the average of the three measured strains was reported. In addition, the beam deflection at the midspan was measured by two linear variable differential transducers (LVDTs) during the fatigue test.

The Poisson stochastic process was combined with the associated damage. This method was introduced to estimate the fatigue life of the specimen. The effectiveness of the method was verified by comparing calculated results with experimental results. The fatigue life of the specimen was estimated as follows:

(1) According to the S-N curve of the epoxy asphalt mixture, the fatigue limit N of the material under various stress load amplitudes could be derived. The homogeneous Poisson strength coefficient of stress loads at all levels was obtained through the calculation of Equation (3): $\lambda_{1}=0.317$, $\lambda_{2}=0.251$, $\lambda_{3}=0.128$, $\lambda_{4}=0.259$.
(2) Based on the distribution relationship of random loads in the test samples, the mean value and variance of each level of load could be calculated, respectively, as shown in Table 1. Then, the values of the parameter p, q in the inhomogeneous Poisson strength function can be solved $\beta \left(i\right)$ due to all levels of loads according to Equation (10).
(3) According to Equation (11), the equivalent amplitude of loads at all levels at any time t could be deduced.
(4) The cumulative fatigue damage values of the material during sample fatigue failure were calculated according to Equation (15). The parameter values calculated through the above steps were replaced with Equation (15) for integral calculation, and the number of test cycles was 1.7456 × 10^7^ times. In other words, upon fatigue failure, the cumulative damage to the sample material is shown in Table 1.

When fatigue failure occurs in the specimen, the cumulative damage to the material should be D=1. The fatigue damage value was calculated by Miner’s linear fatigue damage theory, and the cumulative damage D=0.42 was obtained with an error of 58%. According to the fuzzy fatigue damage theory, with the normal distribution function as the membership function, the error is 29% and D=0.71. According to the method described here, the error is 14% and D=0.86. It can be seen that the combination of the Poisson stochastic process with associated damage theory allows interaction effects between loads during variable-amplitude loading. Thus, the fatigue life estimation of materials is closer to the actual situation.

## 5. Estimated Fatigue Life of Epoxy Asphalt Steel Bridge Deck Pavement

### 5.1. Stress Ladder Load Spectrum of Steel Bridge Deck Pavement

**Figure 4:**
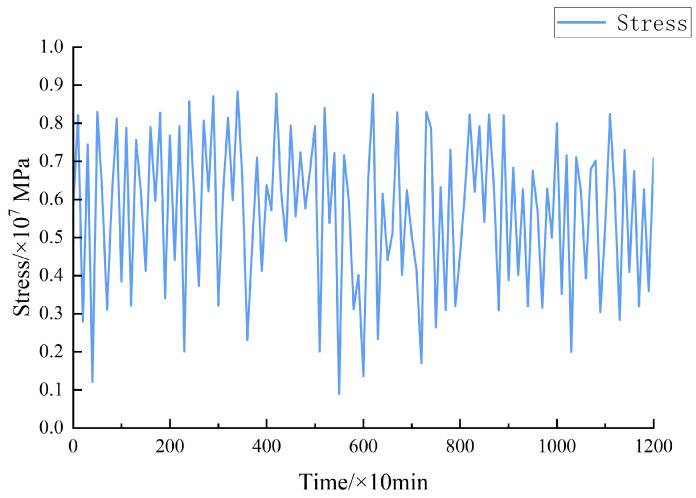
Sample stress–load spectrum.

The objective of this paper is to examine the Sutong Yangtze River Bridge and driving speed. By calculating the measured load, the stress load data of the epoxy asphalt steel bridge deck pavement could be obtained. It has been plotted as a stress–load spectrum, as shown in Figure 4 [35]. Since it is difficult to analyze the continuous loading spectrum, the continuous loading spectrum was converted to an equivalent stepped curve after hierarchical processing for program-controlled loading. The ladder spectrum could be divided into ten loading stages, according to the non-equal interval method. The proportional coefficients of each stage of load and maximum load were 1.000, 0.950, 0.900, 0.850, 0.725, 0.650, 0.575, 0.425, 0.275, and 0.125. In each load stage region, the load amplitude changes were small, and the influence of the interaction between loads on the life estimation was not considered. By using Miner’s equivalent rules, the equivalent stress amplitude of each level of load borne by the steel bridge deck pavement could be calculated.

The equivalent stress amplitude of all levels of loads can be obtained as follows:

(17) $$\sigma_{iD}={\left(\int_{\sigma_{\min}}^{\sigma_{\max}}f\left(\sigma \right)\sigma_{i}^{m}d\sigma \right)}^{\frac{1}{m}}$$

where $\sigma_{iD}$ is the equivalent stress amplitude of all levels of loads; m is the material constant, determined by the test; $f\left(\sigma \right)$ is the distribution density function of the load; $\sigma_{\min}$ is the minimum value in all levels of load-interval; $\sigma_{\max}$ is the maximum value at all levels of load interval.

The load on steel bridge deck pavement satisfies Weibull distribution, and its density function is

(18) $$f\left(\sigma \right)=\frac{a}{b}{\left(\frac{\sigma -c}{b}\right)}^{a-1}\exp\left(-{\left(\frac{\sigma -c}{b}\right)}^{a}\right)$$

where σ is the load stress amplitude of steel bridge deck pavement; a indicates the shape parameter, reflecting the load distribution shape; b is the scale parameter, representing the overall level of load; c is the position parameter, indicating the minimum value of the load.

**Figure 5:**
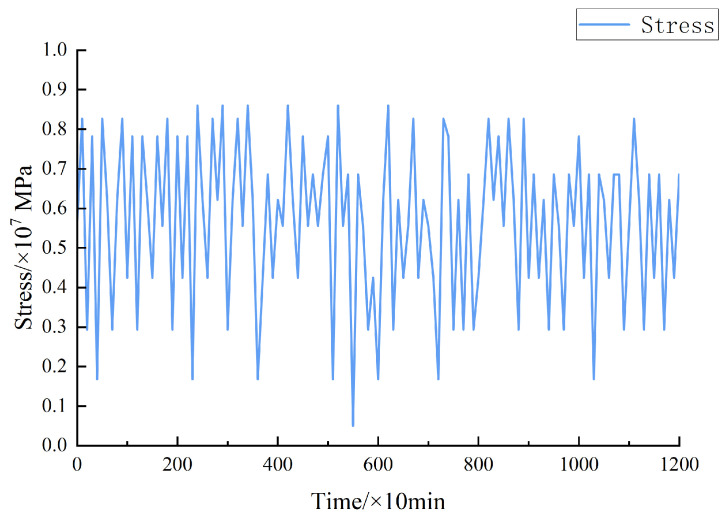
Stress ladder spectrum.

They were calculated according to Equations (17) and (18), and the load spectrum was recomputed. There is an equivalent stress amplitude at all levels of load in Table 1 and the stress ladder load spectrum of steel bridge deck pavement in Figure 5.

### 5.2. Poisson’s Random Process Model Parameters of Steel Deck Asphalt Pavement

The ratio of the occurrence times at all levels of loads to the current cycle time is 0~1 under the current cycle time, so u=0 and ν=1. The expected value $\mu_{x}$ and variance $\sigma_{x}^{2}$ can be determined by the calculation of the sample data. By substituting the expected and variance values into Equation (10), the values of the stress parameters p and q at all levels could be calculated, as shown in Table 4.

### 5.3. Equivalent Load and Fatigue Performance Curve of Steel Deck Asphalt Pavement

At the time t, all levels of load have a certain probability of occurring. After a weighted equivalent treatment of the time t load, the time-equivalent load value could be obtained.

(19) $$\sigma_{e}\left(t\right)=\sum \sigma_{i}P_{i}\left(t\right)=\sigma_{1}\lambda_{1}\left(t\right)+\sigma_{2}\lambda_{2}\left(t\right)+\cdots +\sigma_{8}\lambda_{8}\left(t\right)$$

As a composite material, there was no obvious fatigue limit in the asphalt pavement material. The S-N curve continued to downtrend after cycles of 10^7^ times under small stress. By replacing the S-N curve with the empirical formula, the cycle times $N_{e}\left(t\right)$ of the equivalent stress at all levels were calculated.

(20) $$\alpha \sigma_{e}\left(t\right)+\mathrm{lg}N_{e}\left(t\right)=b$$

where, $\sigma_{b}$ is the static strength of the material, $\sigma_{b}/B=9.88$, α=1/B; $\sigma_{e}\left(t\right)$ and $N_{e}\left(t\right)$ indicate the corresponding stress level and the number of cycles during failure. Take the asphalt mixture material $\sigma_{b}=0.3231\;\mathrm{MPa}$, stress ratio r=0.45, the conditional fatigue limit of 0.5804 MPa. Substituting the obtained data into Equation (20), the value of cycles of equivalent stress at each moment can be obtained.

### 5.4. Comparison of Calculation Results and Precision

Based on the above studies, the total damage to asphalt pavement on steel bridge decks could be derived as follows:

(21) $$D=\int_{0}^{t^{\prime }}\left\{\frac{1}{N_{e}\left(t\right)}+{\left(-1\right)}^{H}\left[1-\exp{\left(-\left(\frac{\sigma_{e}\left(t\right)-a}{b}\right)\right)}^{2}\right]\frac{1}{N_{e}\left(t\right)}\right\}dt$$

When the total damage value reaches 1, i.e., D=1, fatigue failure occurs on asphalt pavement. Therefore, as a condition D=1, the parameters calculated above were added at the same time, and the inverse solution of Equation (21) was obtained. By substituting the material parameters into Equation (21), the theoretical fatigue life expectancy of the bridge deck pavement can be obtained for different loading levels. The calculated results are listed in Table 5.

The total cycle time of steel bridge deck pavement was $t^{\prime }=19,023,927$ when fatigue failure occurred, meeting the requirement for the design life of steel bridge deck pavement (≥12 million times) [34]. Compared with the experiment, the accuracy error is 4.91%, which is smaller than that of the M-H model [35]. The reason for this is that the effect of load interaction on fatigue damage has not been fully considered.

## 6. Conclusions

In this paper, a fatigue life prediction model for the layered asphalt concrete pavement structure on the steel bridge deck is established based on the inhomogeneous Poisson stochastic process, and the feasibility of the model is verified by fatigue tests. The following findings were obtained:

(1) The Poisson stochastic process theory was used to model and analyze the occurrence probability of loads at each moment. The mathematical model of load inhomogeneous Poisson strength function parameters was established by using the incomplete beta function, and thus the problem of path acquisition for the load micro-amplitude variation process has been addressed. The fatigue design of steel bridge deck asphalt pavement with certain accuracy was effectively carried out.
(2) The Poisson stochastic process and associated damage theories were combined to analyze and solve problems from the microscopic variable amplitude process of load. The influence of the interaction between loads on fatigue damage has been fully considered. The results showed that the fatigue life under random load could be more accurately estimated by this method than others. Compared with the experiment, the accuracy error is 4.91%, meeting the needs of engineering design.
(3) The parameters involved in the fatigue design mode could be calculated and analyzed by themselves according to the stress level of the variable amplitude load to avoid excessive parameters. This method can be directly applied to address practical problems with the steel bridge deck pavement.
(4) The higher the load levels, the more obvious the effects on the stress ratio. The fatigue design of the model can well consider multistage loading sequence effects and the environment. In the future, to avoid only considering the loading sequence effects due to the resulting insufficient accuracy of reason analysis, more reliable analysis results should be given to ensure the effectiveness of fatigue analysis and the design of asphalt pavement structures.

It is worth explaining that only epoxy asphalt is considered in this paper, and the rest of the asphalt material for pavement needs to be further studied in the future.

## Figures and Tables

**Table 1:** Design of mixture proportions in the experiment.

Types	Percentage of Particle Mass Passing through Different Sieve Holes (Square Sieve/mm) (%)										Asphalt Content (%)
	16	13.2	9.5	4.75	2.36	1.18	0.6	0.3	0.15	0.075	
EA		100	95–100	65–85	50–70	-	28–40	-	-	7–14	6.5

**Table 2:** Pavement performance of asphalt concretes.

Performance	Test Condition, Unit	EA	Requirement [34]
Void fraction	/%	2.2	≤1.5
Dynamic stability	60 °C, 0.7 MPa, /round/mm	12,600	≥800
	70 °C, 0.7 MPa, /round/mm	9000	≥300
Ultimate bending strength	−15 °C, 1 mm/min, /MPa	18.3	≥10
Ultimate bending strain	−15 °C, 1 mm/min, /10^−3^	2.74	≥2
Indirect tensile strength	25 °C, /MPa	5.39	-
Indirect tensile strength after Freeze–thaw Cycle	/MPa	4.15	-
Linear contraction coefficient	15~−15 °C, /10^−5^ °C^−1^	1.52	≤3.00

**Table 3:** Test parameters of random loading of the four-stage load. is the mean of x, and $\sigma_{x}$ is the variance of x.

$\sigma_{i}$/MPa	$\mu_{x}$	$\sigma_{x}$	Distributed Parameters		Test Life /×10^7^	Number of Test Cycles				Calculation of Cumulative Fatigue Damage		
			p	q		/×10^7^				Miner	M-H	This Paper
0.85	0.317	0.015	3.91	11.25								
0.70	0.251	0.016	2.46	13.33	3.2732	0.6756	0.6381	0.563	1.3888	0.42	0.71	0.86
0.65	0.128	0.008	5.11	16.16								
0.55	0.259	0.013	6.32	9.24								

Note: M-H is Manson–Halford [35].

**Table 4:** Load parameters at all levels.

Level	The Upper and Lower Limit of Load/MPa	Equivalent Load/MPa	Frequency	Probability	$\mu_{x}$	$\sigma_{x}^{2}$	p	q
1.00	0.8327–0.8876	0.7245	52	0.0433	0.0433	0.0002	4.8662	93.2657
0.950	0.8205–0.8327	0.7149	100	0.0833	0.0833	0.00045	18.3859	86.2846
0.900	0.7438–0.8205	0.6526	138	0.1150	0.1150	0.0068	12.9271	48.5651
0.850	0.6281–0.7438	0.5718	305	0.2542	0.2542	0.0027	18.9053	35.4544
0.725	0.6149–0.6281	0.4223	268	0.2233	0.2233	0.0059	6.9965	30.7919
0.650	0.4978–0.6149	0.3868	154	0.1283	0.1283	0.0013	5.4437	55.1447
0.575	0.3519–0.4978	0.3033	102	0.0850	0.0850	0.0016	6.3233	22.8270
0.425	0.2356–0.3519	0.1924	51	0.0425	0.0425	0.0044	0.1972	5.3709
0.275	0.1012–0.2356	0.1129	23	0.0192	0.0192	0.0019	0.1886	11.8640
0.125	0–0.1012	0.0558	7	0.0058	0.0058	0.00016	0.1556	34.2627

**Table 5:** Comparison between the test loading times and theoretical calculation results.

Load Level (kN)	Exp.	Equation (21)	Difference (%)	M-H Model	Difference (%)
12	>30,000	773,414,253	-	78,041,000	-
16	18,133,000	19,023,927	4.91	20,189,000	11.34

## Data Availability

The data presented in this study are available upon request from the corresponding author.

## References

[1] Wolchuk R. (2007). Orthotrope Fahrbahnplatte-Entwicklungen und Möglichkeiten für die Zukunft. Stahlbau.

[2] Seyed H.G., Ji Y.L. (2021). Reliability-based indicator for Post-earthquake traffic flow capacity of a highway bridge. Strusafe.

[3] Huang W., Minshan P., Liu X., Wei Y. (2020). Design and construction of super-long span bridges in China: Review and future perspectives. Front. Struct. Civ. Eng..

[4] Yi X., Wong Y.D., Chen H., Fan Y., Yang J., Huang W. (2023). Utilization of dry-method styrene-butadiene-styrene and epoxy polymer to enhance the aged asphalt binder: Properties evaluation and cost discussion. J. Clean. Prod..

[5] Losa M., Di Natale A. (2012). Evaluation of representative loading frequency for linear elastic analysis of asphalt surfacing. Transp. Res. Rec..

[6] Yin C., Zhang H., Pan Y. (2016). Cracking mechanism and repair techniques of epoxy asphalt on steel bridge deck surfacing. Transp. Res. Rec..

[7] Rout M.K.D., Sahdeo S.K., Biswas S., Roy K., Sinha A.K. (2023). Feasibility Study of Reclaimed Asphalt Pavements (RAP) as Recycled Aggregates Used in Rigid Pavement Construction. Materials.

[8] Zhang L., Wang W., Lu Q., Chen X. (2013). An innovative approach to determine deck surfacing modulus and interfacial slip stiffness based on a composite beam model. Constr. Build. Mater..

[9] Kim T., Baek J., Lee H., Lee S. (2014). Effect of surfacing design parameters on the behavior of orthotropic steel bridge deck surfacings under traffic loading. Int. J. Surf. Eng..

[10] Kainuma S., Jeong Y.S., Ahn J.-H., Yamagami T., Tsukamoto S. (2015). Behavior and stress of orthotropic deck with bulb rib by surface corrosion. J. Constr. Steel Res..

[11] Bocci E., Graziani A., Canestrari F. (2015). Mechanical 3D characterization of epoxy asphalt concrete for surfacing layers of orthotropic steel decks. Constr. Build. Mater..

[12] Hornyak N., Crovetti J.A. (2009). Analysis of load pulse durations for Marquette interchange instrumentation project. Transp. Res. Rec..

[13] Xia T. (2015). Study on the applicability of Miner’s law under multiaxial 2-stage step loading spectra with consideration of material dispersion. J. Mech. Eng..

[14] Medani T.O., Liu X., Huurman M., Scarpas A., Molenaar A. (2010). Characterisation of surfacing materials for orthotropic steel deck bridges. Part 1: Experimental work. Int. J. Surf. Eng..

[15] Kozy B.M., Kozy B.M., Connor R.J., Paterson D., Mertz D.R. (2010). Proposed revisions to AASHTO-LRFD bridge design specifications for orthotropic steel deck bridges. J. Bridge Eng..

[16] de Freitas S.T., Kolstein H., Bijlaard F. (2017). Fatigue assessment of full-scale retrofitted orthotropic bridge decks. J. Bridge Eng..

[17] Liu W., Xu S., Li Q. (2012). Experimental study on fracture performance of ultra-high toughness cementitious composites with J-integral. Eng. Fract. Mech..

[18] Campbell F.C. (2012). Fatigue and Fracture: Understanding the Basics.

[19] Wang X. (2008). Fatigue life estimation method based on fuzzy theory. China Mech. Eng..

[20] Awed A., Kassem E., Masad E., Little D. (2015). Method for predicting the laboratory compaction behavior of asphalt mixtures. Mater. Civil Eng..

[21] Gao P., Xie L. (2010). Reliability analysis of parts based on the load—Strength interference model. China Mech. Eng..

[22] Saboo N., Kumar P. (2015). Optimum blending requirements for EVA modified binder. Int. J. Surf. Res. Technol..

[23] Delgadillo R., Bahia H.U., Lakes R. (2012). A nonlinear constitutive relationship for asphalt binders. Mater. Struct..

[24] De Domenico D., Messina D., Recupero A. (2021). A Combined Experimental–Numerical Framework for Assessing the Load-Bearing Capacity of Existing PC Bridge Decks Accounting for Corrosion of Prestressing Strands. Materials.

[25] Bae J.H., Hwang H.H., Park S.Y. (2019). Structural Safety Evaluation of Precast, Prestressed Concrete Deck Slabs Cast Using 120-MPa High-Performance Concrete with a Reinforced Joint. Materials.

[26] Zhou X., Zhao G., Tighe S., Chen M., Wu S., Adhikari S., Gao Y. (2020). Quantitative comparison of surface and interface adhesive properties of fine aggregate asphalt mixtures composed of basalt, steel slag, and andesite. Mater. Struct..

[27] Pokorski P., Radziszewski P., Sarnowski M. (2016). Fatigue life of asphalt surfacings on bridge decks. Procedia Eng..

[28] Miśkiewicz M., Daszkiewicz K., Lachowicz J., Tysiąc P., Jaskuła P., Wilde K. (2021). Nondestructive methods complemented by FEM calculations in diagnostics of cracks in bridge approach pavement. Autom. Constr..

[29] Alencar G., de Jesus A.M.P., Alencar G., de Jesus A.M.P., Calçada R.A.B., da Silva J.G.S. (2018). Fatigue life evaluation of a composite steel-concrete roadway bridge through the hot-spot stress method considering progressive surfacing deterioration. Eng. Struct..

[30] Zhang Y. (2012). Fault sample simulation based on nonhomogeneous Poisson process and statistical simulation. J. Mech. Eng..

[31] Lu Y., Garrido J. (2004). Doubly periodic non-homogeneous Poisson models for hurricane data. Stat. Methodol..

[32] Lin S. (2003). Parameter fitting optimization of the beta distributions. Instrum. Test.

[33] (2011). Standard Test Material of Bitumen and Bituminous Mixtures for Highway Engineering.

[34] (2017). Specifications for Design of Highway Asphalt Surfacing.

[35] Xu X., Yang X., Yang W., Guo X., Xiang H. (2020). New damage evolution law for modeling fatigue life of asphalt concrete surfacing of long-span steel bridge. Constr. Build. Mater..

